# Generating environmental sampling and testing data for micro- and nanoplastics for use in life cycle impact assessment

**DOI:** 10.1016/j.scitotenv.2022.160038

**Published:** 2023-02-10

**Authors:** Cecilia Askham, Valentina H. Pauna, Anne-Marie Boulay, Peter Fantke, Olivier Jolliet, Jérôme Lavoie, Andy M. Booth, Claire Coutris, Francesca Verones, Miriam Weber, Martina G. Vijver, Amy Lusher, Carla Hajjar

**Affiliations:** a Norwegian Institute for Sustainability Research (NORSUS), Stadion 4, 1671 Kråkerøy, Norway; b International PhD Programme/UNESCO Chair “Environment, Resources and Sustainable Development”, Department of Science and Technology, Parthenope University of Naples, Centro Direzionale, Isola C4, 80143 Naples, Italy; c CIRAIG, Chemical Engineering Department, Polytechnique Montreal, Canada; d Quantitative Sustainability Assessment, Department of Environmental and Resource Engineering, Technical University of Denmark, Produktionstorvet 424, Kgs. Lyngby, Denmark; e CIRAIG, UQÀM/ISE–Institute of Environmental Sciences, Montreal, Canada; f SINTEF Ocean, Trondheim, Norway; g NIBIO Norwegian Institute of Bioeconomy Research, Division of Environment and Natural Resources, Ås, Norway; h Norwegian University of Science and Technology (NTNU), Trondheim, Norway; i HYDRA Marine Sciences GmbH, Bühl, Germany; j Leiden University, Institute of Environmental Sciences, the Netherlands; k Norwegian Institute of Water Research (NIVA), Oslo, Norway; l Department of Biological Science, University of Bergen, Bergen, Norway

**Keywords:** Microplastics, Nanoplastics, Life cycle assessment, Risk assessment, Ecotoxicology, Harmonizing data collection, Monitoring

## Abstract

Ongoing efforts focus on quantifying plastic pollution and describing and estimating the related magnitude of exposure and impacts on human and environmental health. Data gathered during such work usually follows a receptor perspective. However, Life Cycle Assessment (LCA) represents an emitter perspective. This study examines existing data gathering and reporting approaches for field and laboratory studies on micro- and nanoplastics (MNPs) exposure and effects relevant to LCA data inputs. The outcomes indicate that receptor perspective approaches do not typically provide suitable or sufficiently harmonised data. Improved design is needed in the sampling, testing and recording of results using harmonised, validated and comparable methods, with more comprehensive reporting of relevant data. We propose a three-level set of requirements for data recording and reporting to increase the potential for LCA studies and models to utilise data gathered in receptor-oriented studies. We show for which purpose such data can be used as inputs to LCA, particularly in life cycle impact assessment (LCIA) methods. Implementing these requirements will facilitate proper integration of the potential environmental impacts of plastic losses from human activity (e.g. litter) into LCA. Then, the impacts of plastic emissions can eventually be connected and compared with other environmental issues related to anthropogenic activities.

## Highlights

• Monitoring micro- and nanoplastic pollution is usually from a receptor perspective.
• Life cycle assessment (LCA) represents an emitter perspective.
• More comprehensive, harmonised reporting is required from field and lab studies.
• Three levels of reporting requirements are proposed for receptor-oriented studies.
• Micro- and nanoplastic data links to life cycle impact assessment are shown.

## Graphical abstract

**Figure:**
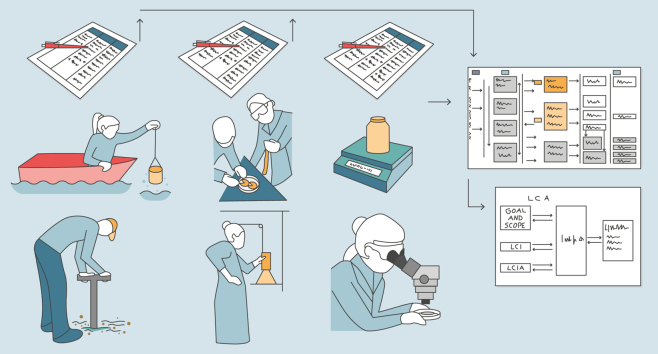

## 1 Introduction

### 1.1 LCA background

Life Cycle Assessment (LCA) is a standardised analysis tool that aims to support decision-making by identifying options or solutions, which are associated with the lowest potential environmental impacts. After a scoping phase, LCA consists of a Life Cycle Inventory (LCI) analysis phase, compiling all resources extracted from, and pollutants emitted to, the environment at each life cycle stage of the product/process system studied. Next, the Life Cycle Impact Assessment (LCIA) phase characterizes these inventory flows in terms of potential impacts in multiple categories (such as ecotoxicity, land use and global warming) to facilitate the comparison of alternatives. The LCIA results can be further translated into the potential damages to main areas of protection, such as human health, ecosystem quality and natural resources. There are scientific papers that address parts of the impact assessment model that relates the emission of plastic to the environment into potential impacts or damage in LCIA (Maga et al., 2022; Salieri et al., 2021; Woods et al., 2021). This work is under development, with many data gaps that researchers are working to fill in order to develop characterization factors (CFs). The CFs required for LCIA calculations are not implemented into software tools for LCIA. When using LCA to compare the environmental performance of alternatives including plastics, impacts associated with plastic emissions are often either missing, or shown directly as an inventory flow (i.e. “x kg of plastic emitted as litter”) alongside impact indicators. This absence of LCIA characterization factors results in an underestimation of potential impacts for the alternatives studied, while the inventory flow approach is misleading and does not represent the diversity of potential environmental impacts associated with plastic emissions and how they may compare with other environmental issues.

### 1.2 Marine Impacts in Life Cycle Assessment (MarILCA)

Knowledge gaps which hinder the connection between the impacts associated with plastic emissions and other environmental issues were highlighted by the LCA community in the Medellin Declaration in 2017 (Sonnemann and Valdivia, 2017). As a response, Marine Impacts in Life Cycle Assessment (MarILCA) was launched in late 2018 with the support of the UN Environment Programme and the Forum for Sustainability through Life Cycle Innovation (FSLCI) to address the knowledge gaps presented by industry experts and by international working groups (Boulay et al., 2019; Saling et al., 2020). MarILCA aims to foster the development of impact assessment methods for marine impacts in LCA, with an initial focus on plastic litter, by favouring collaboration, communication, and consensus-based scientific development. It brings together international experts working on the development of impact pathway modelling of different plastic emissions into the environment (MarILCA, 2020).

### 1.3 Risk assessment of plastics in the environment

Plastics are omnipresent in marine ecosystems and international expert teams are currently working to describe and estimate the magnitude of exposure and impacts (Rochman et al., 2016; SAPEA, 2019; VKM, 2019). Many scientific studies have investigated emission values related to sources and quantified the presence of plastic pollution in the environment (GESAMP, 2016). Micro- and nanoplastics (MNP) are considered to be the form of plastic pollution with the potential for eliciting the greatest impacts. Defining toxicity values that indicate harmful levels of MNPs on ecosystems is an important part of risk assessment (RA). Examples of RA carried out regarding MNPs include Burns and Boxall (2018), Everaert et al. (2018) and Jacques and Prosser (2021). However, numerous studies have highlighted significant data needs to be able to define these harmful exposure levels (Gouin et al., 2019; SAPEA, 2019; VKM, 2019). A combination of environmental sampling (exposure levels) and laboratory testing (impacts) is important for providing data for RA, such data being frequently used in the development of LCIA toxicity models. In this study, the authors describe the links between these data requirements and how comprehensive data provision from environmental sampling and laboratory testing for RA purposes can make more of the MNP data valuable for the LCA community.

### 1.4 Microplastics (>1 μm ≤ 5 mm)

Microplastics (MPs) are a diverse suite of polymer-based contaminants that vary greatly in morphology, chemical properties, texture, colour, density, and size (Rochman, 2016). A variety of organisms representing various habitats, trophic levels, sizes, feeding mechanisms and behaviours, are exposed to MPs (Kukkola et al., 2021; Walkinshaw et al., 2020). Factors such as exposure route and life stage also play a key role in organism-MP interactions and potential impacts. Studies investigating the physiological impacts of MPs on marine organisms demonstrate that interactions between biota and MPs can include ingestion, egestion and, for smaller particle sizes, uptake, accumulation, and tissue transfer (Gouin et al., 2019). Subsequently, a wide range of effects resulting from these different types of interactions have been reported, including stress, adverse impacts on fitness and sometimes even mortality (Cong et al., 2019; Rehse et al., 2016; Sussarellu et al., 2016; Tussellino et al., 2015; Zhang et al., 2017). Particles that are large relative to the size of an organism can block the digestive tract, causing pseudo satiation and diminishing the uptake of food (Weis, 2019). In addition, some entanglement effects have been observed for the smallest organisms, including the entrapment of MPs in the appendages of zooplankton or adsorption of MPs on the surface of microalgae (Bergami et al., 2016; Zhang et al., 2017).

Despite many studies on the physiological impacts of MPs on biota, there remains a current lack of comprehensive understanding regarding the toxicity mechanisms driving the observed effects. For example, many studies on MP effects do not acknowledge the chemicals associated with plastics when investigating effects; the analytical techniques carried out do not always involve a chemical analysis (Lusher et al., 2021). Furthermore, the data used to assess risks are often related only to polymeric compositions or sizes of plastic particles rather than an additional investigation regarding the chemical additives (Capolupo et al., 2020; Koelmans et al., 2017). Plastic-associated chemicals include residual monomers and production chemicals, as well as additives specifically added to plastics to provide specific properties to the material for improved performance, including plasticisers, stabilisers, antistatic agents, and other chemical compounds (Hahladakis et al., 2018). There is also debate about the importance of chemicals that have adsorbed to the MP surface from the surrounding environment, which is often referred to as a vector effect (Koelmans et al., 2016; Sørensen et al., 2020; Syberg et al., 2015; Woods et al., 2021). The longevity of plastic means that there could be adverse impacts occurring for multiple recipient organisms in a cascade over the remaining lifetime of the plastic emission, including the release of plastic-associated chemicals as degradation proceeds (Campanale et al., 2020; Hale et al., 2020; Sørensen et al., 2021).

### 1.5 Nanoplastics (≤1 μm)

MPs and nanoplastics (NPs; defined herein as ≤1 μm) share similarities in terms of origin (e.g. formed through use, or through naturally occurring UV, mechanical and biological degradation processes in the environment), polymeric diversity and general morphological characteristics (Lusher et al., 2017). As the size of plastic particles decreases into the nano range (Mitrano et al., 2021), the extraction and analytical challenges increase and the environmental consequences differ due to changes in transport properties, the way they interact with light and colloids, aspect ratio, bioavailability, and the time frame for additives to diffuse (Gigault et al., 2021). While a number of studies have demonstrated the formation of NPs in the laboratory (Lambert and Wagner, 2016; González-Pleiter et al., 2019; Menzel et al., 2022), the challenges associated with their isolation, identification and quantification mean that only five studies had attempted to determine NP levels in environmental samples as of 2021 (Cai et al., 2021). This work is currently advancing (Abdolahpur Monikh et al., 2021a, Abdolahpur Monikh et al., 2021b) and further studies are expected. Differences in the physicochemical characteristics of NPs relative to MPs can impact their environmental fate and possible physiological effects on biota and humans (Gigault et al., 2021; Noventa et al., 2021). Therefore, these fundamental differences must be acknowledged in the data acquired for LCIA and RA to ensure an accurate analysis is carried out.

## 2 Methods

The current body of literature indicates that researchers in RA and LCA have attempted to draw connections between the two methodologies for >20 years but have faced difficulties due to their different objectives (Linkov et al., 2017). From the LCA perspective, the inclusion of a new emission, such as MNP, will first require establishing the necessary characterization factors that represent the relationship between the emitted quantity of MP or NP to human or ecotoxicological impact categories (Linkov et al., 2017). Furthermore, to establish a complete LCIA characterization factor, it will be necessary to link a fate model of plastic waste which quantifies residence times and degradation rates of plastic debris in the relevant environmental compartment to improve the emerging work on fate factors (Maga et al., 2022; Woods et al., 2021). In this publication we link the MarILCA framework to environmental sampling and laboratory testing. The proposed guidelines developed by the authors will enable the acquisition of MNP data which will address data needs of both RA and LCA. These guidelines were developed through interdisciplinary collaboration among RA experts, LCA experts, scientists focussing on monitoring, and ecotoxicology experts.

### 2.1 The MarILCA Framework and links to environmental sampling and laboratory testing

**Fig. 1:**
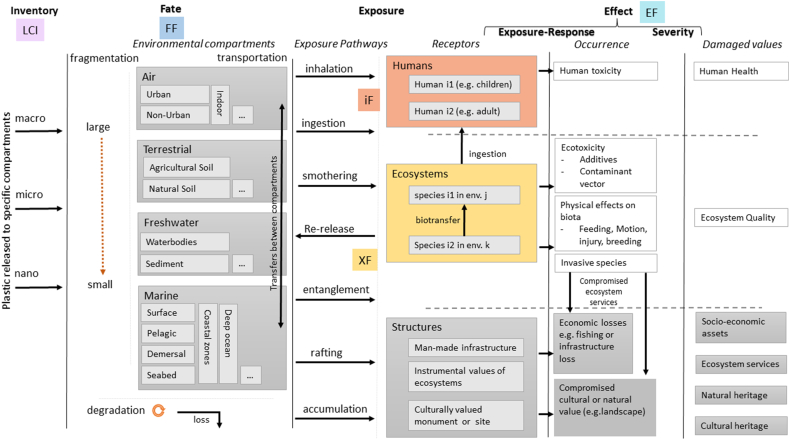
The MarILCA Framework adapted from Woods et al. (2021). The colour-coding is used to highlight the different parts of the LCIA impact pathways (Eqs. (1), (2)) and links with the colours used in Fig. 2, Fig. 3. FF - fate factor, XF - ecosystem exposure factor, iF - human intake fraction and EF - effect factor.

MarILCA developed an impact pathway framework to detail the cause-effect chains identified and associated with environmental plastic emissions to be integrated in LCIA (Woods et al., 2021). This framework is shown in Fig. 1.

As LCA is focused on a product or service value chain, LCA analysts track the plastic flow. LCA can be described as representing an “emitter perspective”; whereby a product and related environmental impacts on multiple ecosystems and locations are followed until its constituent materials reach the end of their lifetime. The emitter perspective can be used to evaluate the environmental impacts of life cycle emissions of one or more given product systems. Built up from this perspective, the MarILCA framework details the steps involved in the LCIA of plastic emitted to the environment (Fig. 1). Plastic is emitted as either macro-, micro-, or nanoplastic, as reported in the inventory (LCI). It undergoes fragmentation and degradation as it passes through or accumulates in different environmental compartments, as characterized by the Fate Factor (FF). The ecosystem exposure factor (XF) characterizes exposure occurring through several exposure routes and pathways. For human health, the intake fraction (iF) combines fate and exposures to give the fraction of the plastic emitted that is taken in by humans. The effect factor (EF) combines exposure-response and severity to yield damages in human health and ecosystem quality.

Environmental sampling and laboratory testing, which provide crucial data for the LCIA models to be developed, is often designed with a receptor perspective in mind as it usually aims to safeguard habitats, or organisms in given habitats. The receptor perspective looks at exposure of a given ecosystem at a given location. This is different to the emitter perspective (that is applicable to LCA), but the data used to derive factors for both perspectives are often similar, which will be expanded on further. The colour-coding added to the framework in Fig. 1 is used to identify and detail the data and information needed to populate and operationalize this proposed framework.

LCIA calculations are shown by Eq. (1) for human toxicity and Eq. (2) for ecotoxicity:

(1) $${\mathrm{CF}}_{\mathrm{human toxicity}}=\mathrm{FF}\times \mathrm{XF}\times \mathrm{EF}=\mathrm{iF}\times \mathrm{EF}$$

(2) $${\mathrm{CF}}_{\mathrm{ecotoxicity}}=\mathrm{FF}\times \mathrm{XF}\times \mathrm{EF}$$

This framework for plastics toxicity follows the scientific consensus model USEtox that has been developed for assessing impacts of chemicals (Rosenbaum et al., 2008). FF (fate factor) has units of kg in compartment per kg emitted/d and denotes the environmental residence time (d). For human toxicity, XF (exposure factor) is expressed in kg intake/d per kg in compartment. iF is the human intake fraction which combines human exposure and fate factors and is expressed in kg intake/kg emitted. Combining it with an EF (effect factor for human toxicity) expressed in cases/kg intake, yields CFs (characterization factors for human toxicity) in cases/kg emitted, with ‘cases’ reflecting cumulative human population incidence risk for a given effect type (e.g. cancer).

The human toxicity effect factor itself is derived from data that are often produced in a RA context, and calculated as a statistical population response level divided by the corresponding chronic Effect Dose, e.g. ED50 for a response of 50 % above the background as used in USEtox until version 2.12 for human toxicity. USEtox is currently being updated, based on global recommendations from the UNEP-hosted Global Guidance on Life Cycle Impact Assessment Indicators (GLAM) project (Fantke et al., 2021). The update for human toxicity includes (i) the evaluation of chemicals in consumer products (in addition to environmental emissions) based on Fantke et al. (2016) and (ii) the determination of the EF as 0.1/ED10, with ED10 (Effective Dose with a 10 % effect level above the background) being based on a stochastic extrapolation from different human and animal points of departure for human toxicity, such as chronic animal in-vivo data (Aurisano et al., 2023). Both updated approaches for deriving human toxicity and ecotoxicity EFs reflect more environmentally relevant chemical exposure levels in addition to making use of a wider range of chronic effect data (Fantke et al., 2018a, Fantke et al., 2018b; UNEP, 2019) and accounting for non-linear dose-response.

For ecotoxicity, XF is expressed in kg bioavailable/kg in compartment, and EF is expressed in PAF·m^3^/kg bioavailable, yielding a CF in PAF·m^3^·d/kg emitted. The effect factor is itself derived from data that are often produced in a RA context, and is calculated as a fraction of species *x* that is potentially affected (PAF) divided by the corresponding Hazard Concentration (HC*x*) at which this fraction of species is affected:

$$\mathrm{EF}=x/\mathrm{HC}x\;\text{with}\;\mathrm{two}\;\text{main options}:\mathrm{EF}=0.5/\mathrm{HC}50_{\mathrm{EC}50},\text{or}\;\mathrm{EF}=0.2/\mathrm{HC}20_{\mathrm{EC}10}$$

USEtox presently recommends the use of chronic chemical toxicity data to generate the Hazard concentration at which 50 % of the species are affected above their individual EC50 levels (HC50_EC50_), calculating the log HC50 as the average of all chronic log EC50s per chemical. Chronic toxicity EC50s are often unavailable, in this case a generic acute-to-chronic ratio of 2 is applied, which has been re-confirmed as average acute-to-chronic ratio in more recent studies (Aurisano et al., 2019). Furthermore, a minimum of three species coming from each of three different taxonomic groups (algae, invertebrates and fishes) is currently recommended for the HC50_EC50_ (Müller et al., 2016). For ecotoxicity, the update recommended by GLAM in its present implementation phase uses HC20_EC10_ values (Hazardous Concentration for 20 % of the species) based on chronic EC10-equivalents (Effect Concentration measured as 10 % effect) derived from various test types (López i Losada et al., 2020; Owsianiak et al., 2022).

There are several studies that derive species sensitivity distributions (SSDs) in order to obtain different endpoints (e.g. PNEC) than those needed for LCIA. Lavoie et al. (2021) harvested data from ecotoxicity studies of virgin MNPs relevant for the marine compartment and used them to create the species sensitivity distribution curves required to obtain HC50s of EC50, as a first contribution to the MarILCA framework (Lavoie et al., 2021). The data was then analysed to see whether it was possible to derive different relationships between toxicity or physical effects, and property parameters such as polymer type, size and morphology. However, statistical analysis of the literature data could not detect any significant difference in effect for any of these property parameters. Thus, Lavoie et al. (2021) currently recommend using a generic effect factor (which does not distinguish between polymer type, size and morphology) combining many different MNPs to account for the physical effect induced by the presence of MNPs in aquatic environments (Lavoie et al., 2021). They also provide a set of EFs for a variety of subclasses of MNPs, such as micro, nano, spheres, polyethylene, polystyrene, and others (Lavoie et al., 2021). SSD curves have also been developed for MPs and NPs in water and soil from an RA perspective (Burns and Boxall, 2018; Jacques and Prosser, 2021), these could be developed into effect factors following the approach of Lavoie et al. (2021).

For impacts from NMPs other than toxicity (e.g. physical effects) units will be different, the conceptual structure of the CFs as shown in Eqs. (1), (2) will, however, remain typically the same.

More and better documented ecotoxicity data for different MNPs (polymer type, morphology and size) is needed in order to facilitate possible differentiation between toxicity effect, physical effect and different sub-groups of MNPs.

In LCIA, the fate factor (FF) links the emission of a certain pollutant to the mass or concentration in the environment (Jolliet et al., 2006). It is expressed in [kg in compartment per kg emitted/d, which can be simplified to d] (Rosenbaum et al., 2008). FFs are based on multimedia mass balance models that account for multimedia and spatial distribution of pollutants as well as their residence time in environmental compartments (Mackay and MacLeod, 2002; Hauschild and Huijbregts, 2015). In the framework of USEtox, the environment is divided into different compartments and fate factors are developed based on transfer and degradation rates represented in a matrix **K** of constant rate coefficients (Fantke et al., 2017). The corresponding matrix of fate factors is obtained by inverting the **K** matrix as **FF** = **K**^−1^ (Rosenbaum et al., 2007), which is the same for human toxicity and ecotoxicity characterization (Jolliet et al., 2006). Fate factors (FF_i,j_) are interpreted as the mass increase of the considered pollutant in a receiving compartment after an emission to the source compartment (Henderson et al., 2011; Jolliet et al., 2006; Rosenbaum et al., 2007). They can also be obtained by multiplying the fraction transferred from the emission to the receiving compartment and the residence time in the destination compartment (Jolliet et al., 2006). An LCIA fate model for MNPs should therefore account for their residence time within spatially defined environmental compartments and sub-compartments by quantifying their transfer and degradation rates; degradation rate can be derived or estimated from fragmentation and biodegradation rates, among others (Saling et al., 2020; Woods et al., 2021). MPs fate mechanisms are influenced by their physical properties (density, size, shape) and environmental conditions that should be taken into consideration. Currently, fate modelling of MPs in the marine environment is most advanced compared to other environmental compartments. A first proposal was published by Saling et al. (2020) that developed fate factors for MPs based on their degradation and fragmentation rates. A recent approach proposed preliminary fate factors for different types of polymers (EPS and tire and road wear particles TRWP) while considering marine water as a single compartment (Corella-Puertas et al., 2022). Fragmentation from macro- to microplastics, degradation, and sedimentation rates were studied between different scenarios that reflected the need for developing polymer-specific fate factors (Corella-Puertas et al., 2022). A more recent study focused on providing degradation rates for different types of polymers in different environmental compartments (soil, river sediment, marine water, and marine sediments). FFs were then developed by combining degradation rates with transfer rates between environmental compartments, and presented using Germany as an example (Maga et al., 2022). A recent review by Malli et al. (2022) considers transport mechanisms and fate of MPs in estuarine compartments. The difference in the fate of different types of MPs emphasized the need for considering different transport mechanisms in different marine sub-compartments which is ongoing work within MarILCA (Hajjar et al., 2020).

## 3 Results and discussion

Data availability and data quality are often mentioned as limiting factors in the literature focused on incorporating marine litter into both RA and LCA. Among others, VKM and Gouin et al. (2019) describe a severe lack of data, meaning that risk cannot be quantified with the current state of knowledge (Gouin et al., 2019; VKM, 2019). Metadata or other quality data is often lacking in MNP research, the reasons having been described above, namely: (i) lack of harmonisation in sampling, sample processing and analysis, and (ii) inconsistent reporting of data and units (Cowger et al., 2020; Gouin et al., 2019; Koelmans et al., 2020; Pauna et al., 2022; Provencher et al., 2020). It is unsurprising that these issues are problematic given the complexity and diversity of MNP physicochemical properties that influence their fate in the environment. In many cases, critical MNP property descriptors cover a continuum of values, for example density (buoyancy), and size, with properties, such as morphology being difficult to measure empirically (Chubarenko et al., 2016; Kooi et al., 2017; Kooi and Koelmans, 2019; Kowalski et al., 2016; Kukulka et al., 2012; Reisser et al., 2013). In addition to the intrinsic physicochemical properties of MNPs, their behavior and fate is also influenced by a range of extrinsic environmental conditions (water density, wind speed, etc.) and transport mechanisms (aggregation, advection, etc.). Moreover, the fragmentation and degradation rates of individual MNPs also modify their physicochemical properties and therefore affect their fate. Degradation rates are influenced by the surrounding environmental conditions (temperature, light, oxygen concentration, etc.) and if biodegradable, the presence of microbes and nutrients. Such metadata are urgently needed to be able to calculate the lifetime of plastic items and MNPs. For biodegradable plastics, biodegradation rates can be calculated if relevant data points are available for a sufficient number of points in time, otherwise these data can be available in open sources (e.g. as proposed by SAPEA, 2020), if the literature data corresponds to relevant environmental conditions (SAPEA, 2020). Given these complexities, metadata needs to be produced and made accessible, allowing for acceptable comparison of datasets from different studies and to help address the current incompatibility of data (Koelmans et al., 2020). Metadata is important for adequately linking LCIA methods with RA, so that these tools can be used to inform one another.

To facilitate a wider uptake and more impactful use in LCA, the authors have generated data reporting requirements for MNP occurrence, fate and effects data generated during field sampling and laboratory testing.

### 3.1 Data requirements

The data requirements from field studies and laboratory testing for MNPs that would facilitate a successful link to LCIA modelling and data needs are provided in Table 1. This requirements list has three levels of detail: Level 1 describes minimum requirements; Level 2 suggests additional data that can often be readily obtained using standard analytical equipment and methods; Level 3 involves data requests that sometimes require more specific and advanced analytical equipment and methods. The data gathering requirements shown in Table 1 are aimed at scientists and practitioners working with field sampling and laboratory analysis of MNPs.

**Table 1:** Data requirements to facilitate acceptable data acquisition and reporting used in LCA. Level 1 describes minimum requirements, Level 2 suggests additional data that can often be obtained with standard analytical equipment and methods, and Level 3 involves data requests that sometimes require more specific and advanced analytical equipment and methods.

Data needs - field studies		
Aim of empirical data study	Metadata^a^	Data type(s)/units
MNP occurrence and distribution, MNP lifetime (e.g. persistence or degradation rate), and MNP impacts at the ecosystem scale.	**Level 1** 1) location in latitude and longitude 2) date and time of sampling 3) depth of sampling 4) sampling device used a) description b) size c) mesh size (if relevant) 5) conditions of the sampling environment: a) wind speed (knots) b) wind direction (degrees) c) sea state (Douglas scale (0–9) or Beaufort scale (0–12)) d) temperature (°C) e) pH f) oxygen g) salinity h) habitat 6) In the case of effluent sampling (LCI): a) production rate of products from factory or wastewater treatment plant at time of sampling. b) total volume of effluent water being discharged at time of sampling.	**Level 1** **1) Qualitative:** a) polymeric composition: e.g. PET, HDPE, LDPE, PVC, PP, PS, PBS, PBAT b) evidence of fragmentation: yes/no c) morphology: categorised as fragment, pellet, fiber, film, or foam d) colour of particles **2) Quantitative:** a) total mass of particles b) dimensions: nano (≤1 μm), small micro (>1 μm ≤ 1 mm), large micro (>1 mm ≤ 5 mm) **3) Units:** a) SEAWATER/FRESHWATER i) # of MNP/volume water ii) mass MNP/volume water b) SEDIMENT/SOIL i) # of MNP/sediment or soil weight (dry, or wet, if wet weight provide moisture content) ii) mass of MNP/sediment or soil weight (dry, or wet, if wet weight provide moisture content) c) BIOTA i) # of MNP/unit (specify if unit is body mass, biomass or individual – if individual also provide mass of individual; dry, or wet, if wet weight provide moisture content) ii) mass MNP/unit (specify if unit is body mass, biomass or individual – if individual also provide mass of individual; dry, or wet, if wet weight provide moisture content).
	**Level 2** As for Level 1	**Level 2** As for Level 1, plus: **1) Qualitative:** e) biofouling: yes/no f) additives in particles **2) Quantitative:** b) dimensions: ii) small micro: >1 μm ≤ 100 μm, >100 μm ≤ 250 μm, >250 μm ≤ 500 μm, >500 μm ≤ 1000 μm c) mass of particles per size range d) aspect ratio **3) Units:** a) SEAWATER/FRESHWATER iii) size distribution (%) b) SEDIMENT/SOIL: iii) size distribution (%) c) BIOTA: iii) size distribution (%)
	**Level 3** As for Level 2, plus: 7) other chemicals or additives found in samples	**Level 3** As for Level 2, plus: **2) Quantitative:** b) dimensions: all raw sizes listed along with a size distribution e) mass of individual particles f) % monomers within polymers observed **3) Units:** a) SEAWATER/FRESHWATER: iv) MNP surface area (m^2^/g) b) SEDIMENT/SOIL: iv) MNP surface area (m^2^/g) c) BIOTA: iv) MNP surface area (m^2^/g)


Data needs - laboratory analysis		
Aim of empirical data study	Metadata^b^	Data type(s)/units
MNP lifetime (e.g. persistence or degradation rate) or MNP effects (e.g. to determine endpoints associated with toxicity)	**Level 1** 1) state of plastic particles (e.g. virgin, fragmented, and/or biofouled) 2) polymer type(s): e.g. PET, HDPE, LDPE, PVC, PP, PS, PBS, PBAT 3) additives in polymers 4) ecotoxicological information: effect concentration (EC*x*) - species, exposure duration and which effect endpoint (mortality, growth, reproduction, development), effect level and units. 5) MNP lifetime information: a) Test level (lab, tank) i) test material, replicates, test duration, sampling points ii) measured parameters (e.g. CO_2_, CH_4_, O_2_, disintegration, weight loss) and units. b) Test conditions i) matrices used, i.e. soil, freshwater, marine ii) grain size distribution (soil/sediment) iii) nutrient concentration iv) temperature (°C) v) conductivity vi) pH	**Level 1** **1) Qualitative:** a) mass-based dose metric b) polymeric composition: e.g. PET, HDPE, LDPE, PVC, PP, PS, PBS, PBAT c) fragmentation: yes/no d) morphology: categorised as fragment, pellet, fiber, film, or foam e) colour of particles f) BIOTA: presence or absence of significant effects (yes or no) g) BIOTA: direction of effect (up = induction, down = inhibition) **2) Quantitative:** a) total mass of particles b) dimensions: nano (≤1 μm), small micro (>1 μm ≤ 1 mm), large micro (>1 mm ≤ 5 mm) **3) Units:** a) SEAWATER/FRESHWATER i) # of MNP/volume water ii) mass MNP/volume water b) SEDIMENT/SOIL i) # of MNP/sediment weight (dry, or wet, if wet weight provide moisture content) ii) mass of MNP/sediment weight (dry, or wet, if wet weight provide moisture content) c) BIOTA i) # of MNP/individual (also provide mass of individual; dry, or wet, if wet weight provide moisture content) ii) mass MNP/individual (also provide mass of individual; dry, or wet, if wet weight provide moisture content)
	**Level 2** As for Level 1, plus: 6) Additional Information: a) NOEC b) LOEC 5) MNP lifetime information: a) Test level ii) CO_2_, CH_4_, O_2_ emissions iii) disintegration and weight loss	**Level 2** As for Level 1, plus: **1) Qualitative:** h) biofouling: yes/no i) additives in particles **2) Quantitative:** b) dimensions: ii) small micro: >1 μm ≤ 100 μm, >100 μm ≤ 250 μm, >250 μm ≤ 500 μm, >500 μm ≤ 1000 μm c) mass of particles per size range d) aspect ratio e) mass of CO_2_, CH_4_ and O_2_ emissions/mass of plastic tested (degradation studies). f) mass loss/mass of plastic tested (degradation studies). **3) Units:** a) SEAWATER iii) size distribution (%) b) SEDIMENT: iii) size distribution (%) c) BIOTA: iii) size distribution (%)
	**Level 3**	**Level 3** As for Level 2, plus: **2) Quantitative:** b) dimensions: all raw sizes listed along with a size distribution g) mass of individual particles h) % monomers within polymers i) degradation rate **3) Units:** a) SEAWATER/FRESHWATER: iv) MNP surface area (m^2^/g) b) SEDIMENT/SOIL: iv) MNP surface area (m^2^/g) c) BIOTA: iv) MNP surface area (m^2^/g)

a Informed by example subtidal sediment sampling sheets provided by Frias et al. (2018).

b Effects considered in the study should be categorised following levels of biological organization (Galloway et al., 2017).

### 3.2 The importance of implementing the data requirements

Level 1 requirements (Table 1) are the minimum data requirements for RA and LCIA, e.g. the specific location (latitude and longitude coordinates) and depth of the sampling for field studies. This information is important for LCIA CF developers to know the relevance of the data. If it is gathered for a very specific habitat, sampling and environmental conditions will be important in order to ascertain whether and/or how it can be applied to other habitats and regions. Levels 2 and 3 for both field and laboratory data improve resolution and sophistication of the available data for analysis and application. Metadata regarding the environmental conditions and sampling location is important for determining the relevance and correspondence among other existing datasets. MNP concentration data that are not adequately supported by metadata has much lower value from an LCIA and RA perspective. MP reporting guidelines are described as important in order to help ensure no critical information is omitted (Michida et al., 2019; Cowger et al., 2020). These guidelines are recommended as a reference source when carrying out both field and laboratory MNP studies. The checklist provided by therein can guide experimental design and data recording to ensure that all related procedures are thoroughly documented (Cowger et al., 2020). The need for reporting requirements and size specifications of MNPs in seawater samples is also described in Michida et al. (2019) which form part of the G20 recommendations which were prepared with the view of enabling researchers of ocean surface layer microplastic monitoring to adopt similar monitoring protocols and therefore interpret their results with a level of comparability. The development of reporting requirements for other sample matrices, including complex waters (influent and effluent), sediments and soils, are expected to emerge in response to monitoring requirements. By following these requirements, data generated will support LCI and LCIA across the product life cycle and value chain.

Field sampling of aqueous effluent streams (e.g. for a given factory) could be useful for LCI data gathering, if the sample results can be linked to the amount of production occurring at the given factory site the effluent comes from at the time. The data gathered should also include the production rate of the factory (e.g., tonnes of plastic produced per hour, tonnes of plastic products produced per hour or tonnes of recycled plastic produced per hour) and total volume of effluent water (e.g., m^3^/h). This would enable a link between the concentration of MNPs in emitted effluents and the associated product value chains.

It is often difficult to obtain any link to LCI data from general environmental sampling, particularly for MNPs. The small size of the particles makes it essentially impossible to establish a clear product link. If unplanned emissions such as littering are to be accounted for in LCI data for a product system, then sources like Elliott et al. (2018), Peano et al. (2020), and de Sadeleer and Askham (2020) will be used to estimate likely rates of littering for given products in a value chain.

Table 1 also describes datatypes, including units. Where sediment and biota are sampled, it is important to document whether the weights recorded refer to dry weight or wet weight. Either could be used, but it is important that this is stated along with moisture content (if wet weight).

The authors have chosen to include information about number and mass of particles in the data requirements. The density of plastics varies according to the type of polymer and additives that are present. The density of MNPs also varies according to polymer composition but can also be significantly influenced by processes such as biofouling (referred to as biofilm formation in some studies), hetero-agglomeration, weathering, and degradation. In practice, the number of particles is often combined with density values assumed for given polymers in order to calculate the mass. Biofouling can also influence how attractive MNPs are for consumption by organisms in the food web by making MNPs mimic natural food items, thus affecting endpoints such as consumption rates and dose-effect relationships (Vroom et al., 2017; Hodgson et al., 2018). The morphology and size of the MNP particles have been shown to affect the availability and effects they can have (Strungaru et al., 2019; Wang et al., 2022).

An illustrative example of how the data described in Table 1 could be used for LCA would be a plastic recycling plant using washing water that is released into a local river and where a series of effluent and river water samples are taken (e.g. 10 samples of 250 ml over one hour of factory operation). As the effluent and river water samples are collected the metadata described in Table 1 should be recorded, e.g. sampling site location, date and time of sampling, depth of sampling (in this case whether it's an outlet sample, or further out in the river will be important to know), what sampling device was used and the conditions of the sampling environment. These metadata ensure the good practice VKM (2019), Gouin et al. (2019) and Cowger et al. (2020) are asking for (suitability for RA studies). Linking the effluent and river water sample to the metadata needed for LCI purposes requires recording the factory's production rate (e.g. that they are producing 1 t of washed HDPE household plastic packaging per hour) and the total volume of effluent water discharged (e.g. 1.5 m^3^ per hour). If one assumes at the time of sampling, that the production rate was constant, and that the average mass of MNP in the 10 samples (sample volume 250 ml) was 3 g (i.e. 24 g/l), then the amount of MNP emitted per tonne of HDPE recycled is:

(3) $$\frac{\left(\left(\frac{24\;g/l\times 1000\;l/m^{3}}{1000\;g/\mathrm{kg}}\right)\times 1.5\;m^{3}/\mathrm{hr}\right)}{1000\;\mathrm{kg}\;\text{HDPE}/\mathrm{hr}}=0.036\;\mathrm{kg}\;\mathrm{MNP}\;\text{emitted}\;\mathrm{per}\;\mathrm{kg}\;\text{HPDE recycled}$$

If the data requirements from Table 1 are followed, the sample analysis will also include polymeric composition, so the user of the data can see whether the emissions are purely HDPE, or whether there are other plastics and contaminants in the recycling stream. The effect factors currently available are limited and often only address “plastic”, but the polymeric composition will be important when the emerging effect factor work progresses. The physical and chemical properties of different polymers can be quite different, affecting both their fate in an environmental compartment and the effects they can have (Rochman et al., 2016; Lins et al., 2022). When the effect factor knowledge becomes sophisticated enough, the morphology, and size data will directly inform the EF for the MNP emissions from the recycling plant. The colour of the particles may help identify the MNP source if there are several possible plastic sources, polymers, or contaminants.

If the recycling plant's outlet into the river is close to the sea, collecting samples of biota (e.g. blue mussels) from around the river mouth can be done to assess whether the biota are affected by the MNP emissions. It is then important for modelling of the effluent fate to know metadata, such as location and depth of the collected samples (see Table 1). The habitat and substrate where the organisms are collected is important to record (i.e. rocky, sandy, coast, open water etc.), as it impacts the living conditions, food availability and potential exposure. The sampled blue mussels are taken to a lab, the soft tissue is extracted from the shell, then degraded (using a digestive substance that has a minimal effect on the plastic, such as potassium hydroxide or an appropriate enzyme). The digestate can then be fractionated using a series of filters of different pore sizes (starting larger and getting smaller to obtain size ranges and distributions). The results obtained give a snapshot of the amount and types of MNPs present in the given biota. These results can be used to verify the expected sources of MNPs in the given catchment area (as in Pauna and Askham, 2022). For physical effects of microplastic (with the EF from Lavoie et al., 2021) the XF is equal to 1 as it is assumed that the total amount of MNP reaching an aquatic environment is available to organisms. If considering the inclusion of toxic effects of chemical additives, the bioavailability of the relevant toxic chemical would need to be considered to modify the XF accordingly as done in Tang et al. (2022).

In a second example, environmental samples are taken in a specific location to examine the occurrence and distribution of MNPs. The sediment, water-column (three different depths) and surface water are sampled in the Mediterranean Sea. Biota samples in the form of sea cucumbers are also taken. There is no specific focus on sources of MNP emissions in this example and in the specific location. The amounts of MNPs present in the sediment can be used to indicate the temporal presence of MNPs (if depth of sample is sufficient, i.e. ≥ than 2 cm below the sediment surface; exposure over time) and comparing that to the presence of MNPs in the other samples (water and biota) will provide information about what levels of MNP are present in the habitat and species (exposure). The presence of MNPs in the different samples can also be used to validate theoretical fate models (e.g. Kane et al., 2020).

For laboratory studies generating effects data, it is important that the exposure duration and effect endpoint are recorded (specific EC level), including which effect endpoint (e.g., mortality, development). If LCIA is to make use of EC values from laboratory studies (see methods for the relevant LCIA steps where EC values are used) these data are needed in order to reliably use the laboratory study data correctly.

Data requirements for studies aiming to determine degradation rate are included in Table 1. Degradation rates depend on MNPs densities (ρ) and specific surface area (SA) which is linked to their shape and size (Chamas et al., 2020; Corella-Puertas et al., 2022). Specific surface degradation rates are also relevant for determining degradation rates, which are based on the mass loss due to fragmentation/degradation processes. The data described in Table 1 is independent of whether the plastic is defined as biodegradable (for definitions see SAPEA, 2020). The market share of biodegradable plastics is still low and there is some scepticism to adoption of these plastics, due to differences in standardised biodegradability tests and conditions in nature (Hann et al., 2020). It is possible that legal framework conditions (such as bans on single use plastic use in Europe) could lead to broader adoption of biodegradable polymers. There are few public LCA studies of biodegradable and/or compostable plastic products; those studied by Hann et al. (2020) had limitations hampering a full comparison between compostable and fossil counterparts due to limitations on data on compostable products (Hann et al., 2020). Biodegradable plastics and MNPs arising from these are challenging to include in LCAs. How the FF and EF parts of the CF modelling are affected by biodegradability need to be taken into account. This will include consideration of rates of degradation in the relevant environmental conditions, changes in degradation rates over time, and changes in accumulation as degradation occurs. These issues are also relevant for MNPs where the type of plastic is not classified as biodegradable or compostable, however the underlying models are likely to need specific adjustments.

An example of a laboratory study and how this can be linked to the EF in LCIA is a toxicity experiment using *Daphnia magna*. The *D. magna* are kept in glass vessels in a controlled environment (or microcosm). The vessels are spiked with a known dose of MNP particles that needs to be recorded (mass-based dose metric, see Table 1). It is important to record the chemical composition of the MNPs, including additives (as described above, additives can vary from granulate suppliers depending on the intended application of the plastic and how it is to be processed, e.g. HDPE intended for injection moulding to a food contact product will have a different additive mix to HDPE intended for plastic bag production via a film production process; different colours also mean a different additive mix). Observations of the biota and the chosen toxicological endpoints should be recorded as described in Table 1, including the presence or absence of significant effects. Concentrations of MNPs leading to e.g. immobilisation of 10 % of a *D. magna* population (i.e. chronic EC_10_) would provide data for one species needed to derive SSDs for EF calculations according to GLAM recommendations (Owsianiak et al., 2022).

**Fig. 2:**
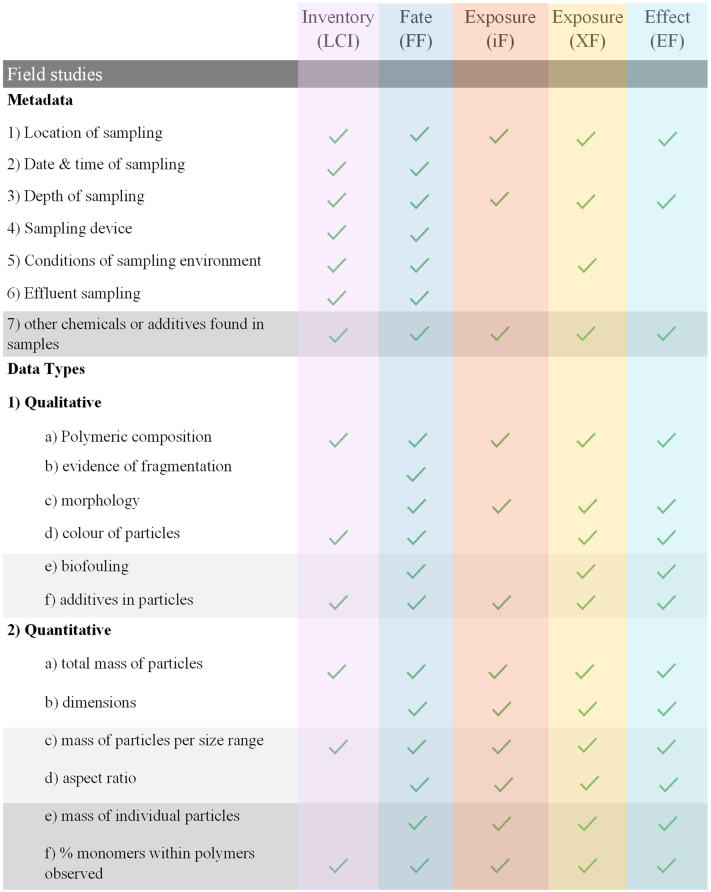
Links between Table 1 and LCIA for field study data collection. The checkmarks indicate that the LCIA category (columns) can be directly informed by the type of data (rows). The shading in the table is in line with the shading of the different levels (1–3) in Table 1.

**Fig. 3:**
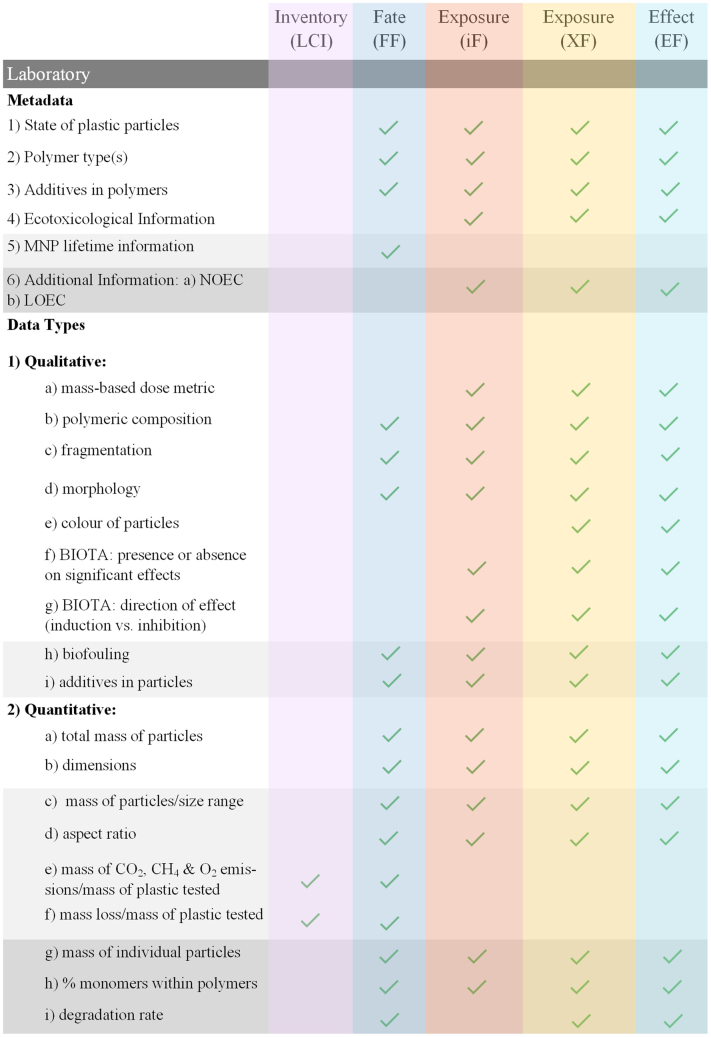
Links between Table 1 and LCIA for laboratory data collection. The checkmarks indicate that the LCIA category (columns) can be directly informed by the type of data (rows). The shading in the table is in line with the shading of the different levels (1–3) in Table 1.

The links between the needs of the MarILCA framework presented in Fig. 1 and the MNP data requirements presented in Table 1 are outlined for field studies in Fig. 2 and for laboratory studies in Fig. 3. As previously described, the CFs used in LCIA are derived from FF, XF (ecotoxicity) or iF (human toxicity) and EF values. Some of the links between the data recording requirements (Table 1) and the LCA data requirements illustrated in Fig. 2, Fig. 3 are described in more detail below.

The field study metadata requirements outlined in Table 1 can inform the FF by contributing data about what types of MNPs, with which characteristics are present in known environmental compartments and locations. Field data is not needed to develop fate models, they can be developed theoretically without field data (e.g. Maga et al., 2022). Field data can however help in validating fate models, or informing specific parts of these models such as degradation, biofouling rates, etc. Such validation could also be based on lab studies. As described in Corella-Puertas et al. (2022), surface area (SA), and density (ρ) are needed to calculate degradation rate. The residence time and the sedimentation percentage are needed to calculate sedimentation rate; degradation rate + sedimentation rate results in the total removal rate of a given polymer. This removal rate provided in kg/(kg*day) can be used as the FF. The iF and XF can be informed by the field study MNP data described in Table 1, as they provide important information about the levels and types of MNP present in the relevant environmental compartments where exposure would occur. Level 1 field study data will inform whether the MNPs will be bioavailable and relevant for intake and exposure. The Level 1 laboratory metadata in Table 1 regarding endpoints would inform the iF, XF and EF, as they focus on endpoints for individuals and populations. Laboratory endpoint metadata can inform FF modelling, as it includes ecosystem endpoints.

## 4 Recommendations/outlook

The current situation, where much MNP data gathered from a recipient perspective is discarded by LCA analysts and LCIA method developers, is very inefficient. The data reporting requirements specified in this paper are beneficial, as they will provide guidance to scientists gathering valuable data in the field, or in the lab. All relevant parties engaged in such data collection should develop and refine protocols based on the reporting requirements outlined here. This will facilitate a step change in the utility of datasets for achieving the aim of incorporating the effects of MNPs into LCA. This will contribute to the proper integration of the potential environmental impacts of plastic litter into LCA and allow for a more adequate comparison of the impacts of plastic products with available alternatives. This will ultimately lead to the ability to connect and compare the impacts associated with plastic emissions with other environmental issues related to anthropogenic activities.

## Nomenclature

CF: characterization factor
d: day
*D. magna*: *Daphnia magna*
EC: effect concentration
EC10: the effect concentration where 10 % of the test organism is affected
EC50: the effect concentration where 50 % of the test organism is affected
ED10: effective dose with a 10 % effect level above the background
ED50: chronic effect dose for a response of 50 % above the background
EF: effect factor
EPS: expanded polystyrene
FF: fate factor
G20: group of Twenty intergovernmental forum comprising 19 countries and the European Union
GLAM: Global Guidance on Life Cycle Impact Assessment Indicators
HC: hazard concentration
HC50: hazard concentration at which 50 % of the species are affected above their individual EC50
HDPE: high density polyethylene
iF: intake fraction
LCA: life cycle assessment
LCI: life cycle inventory
LCIA: life cycle impact assessment
LDPE: low density polyethylene
LOEC: lowest observed effect concentration
MarILCA: Marine Impacts in Life Cycle Assessment
MNPs: micro- and nanoplastics
MP: microplastic
NOEC: no observable effect concentration
NP: nanoplastic
PAF: potentially affected fraction of species
PBAT: polybutylene adipate terephthalate
PBS: polybutylene succinate
PET: polyethylene terephthalate
PNEC: predicted no-effect concentration
PP: polypropylene
PS: polystyrene
PVC: poly vinyl chloride
ρ: density
RA: risk assessment
SA: surface area
SAPEA: the Science Advice for Policy by European Academies consortium
SSD: species sensitivity distribution
TRWP: tire and road wear particles
UN: United Nations
UNEP: United Nations Environment Programme
UNESCO: United Nations Educational, Scientific and Cultural Organization
USEtox: a scientific consensus model endorsed by UNEP's Life Cycle Initiative for characterizing human and ecotoxicological impacts of chemicals
UV: ultraviolet
VKM: Vitenskapskomiteen for mat og miljø (Norwegian Scientific Committee for Food and Environment)
XF: exposure factor

## Author information

The author list has been devised based on the Vancouver Recommendations (2020) promoted as good practice by The Norwegian National Research Ethics Committees. This includes the criteria that the authors have all contributed to the conception or design of the work, analysis, or interpretation of data for the work, as well as drafting or revising it critically for important intellectual content. The order of authorship reflects the contributions of different authors, in the order of greatest to least. Askham, C. was responsible for project administration and supervision of the present work. In addition, Askham, C. and Pauna, V.H. were responsible for the conceptualization, methodology, visualization, and writing – original draft, review & editing. Boulay, A.-M., Fantke, P., Jolliet, O., Lavoie, J., Booth, A., Coutris, C., Verones, F., Weber, M., Vijver, M., Lusher, A.L. and Hajjar, C. contributed through validation, and writing – topic specific contributions to original draft, review & editing.

## CRediT authorship contribution statement

The author list has been devised based on the Vancouver Recommendations (2020) promoted as good practice by The Norwegian National Research Ethics Committees^60^. This includes the criteria that the authors have all contributed to the conception or design of the work, analysis, or interpretation of data for the work, as well as drafting or revising it critically for important intellectual content. The order of authorship reflects the contributions of different authors, in the order of greatest to least. Askham, C. was responsible for project administration and supervision of the present work. In addition, Askham, C. and Pauna, V.H. were responsible for the conceptualization, methodology, visualization, and writing – original draft, review & editing. Boulay, A.-M., Fantke, P., Jolliet, O., Lavoie, J., Booth, A., Coutris, C., Verones, F., Weber, M., Vijver, M., Lusher, A.L. and Hajjar, C. contributed through validation, and writing – topic specific contributions to original draft, review & editing.

## Declaration of competing interest

The authors declare that they have no known competing financial interests or personal relationships that could have appeared to influence the work reported in this paper.

## Data Availability

No data was used for the research described in the article.
